# How objects become psychologically available: in-itself information and informational mediation for theoretical psychology

**DOI:** 10.3389/fpsyg.2026.1885628

**Published:** 2026-09-11

**Authors:** Kun Wu, Jingzhuang Bi, Zhensong Wang

**Affiliations:** School of Humanities and Social Science, Xi'an Jiaotong University, Xi'an, Shaanxi, China

**Keywords:** cognitive generation, information field, information qualities, mind–world relation, philosophical psychology

## Abstract

Psychological science has long examined how subjects perceive, represent, predict, and understand the world. Yet many cognitive theories begin from the assumption that objects are already available to perception or representation, while paying less attention to the prior question of how objects become cognitively accessible before subjective representation. This article addresses this issue through the concept of in-itself information and proposes informational mediation as a conceptual framework for rethinking the mind–world relation. In-itself information refers to the structured disclosure of an object through its states, differences, relations, interactions, and traces prior to a subject’s completed representation or interpretation. Situated, embodied, and predictive accounts have clarified organism–environment coupling, action-oriented cognition, and predictive regulation. The present framework reframes a prior condition often left implicit in these accounts by arguing that objects must become available through informational disclosure before they can be perceived, predicted, represented, or acted upon. On this basis, the article reconstructs the traditional subject–object schema as a subject–information–object relation. A psychological object is understood as a relational outcome generated through information disclosed by objects and the subject’s perceptual, mnemonic, cognitive, and symbolic operations. The framework further suggests that changes in the organization of object-information, especially in technologically mediated and algorithmically structured environments, may alter the conditions under which psychological objects are formed. The article contributes to theoretical and philosophical psychology by clarifying the pre-representational status of objects and by offering a conceptual model for analyzing cognitive access, psychological object formation, and technologically mediated cognition.

## 1 Introduction

The theoretical development of psychological science has always revolved around the fundamental question of how the mind relates to the world. From the subject–object relation in classical epistemology, to the stimulus–response model of behaviorism, and further to the information-processing models of cognitive psychology (Neisser, 1967; Newell and Simon, 1972; Fodor, 1975; Marr, 1982), psychology has continuously sought to explain how the external world enters subjective experience and becomes psychological reality through perception, memory, representation, and judgment. However, different theoretical approaches to cognition presuppose, to varying degrees, that the object can already be contacted, can stimulate the subject, or can be represented or processed by the subject in some way. The question, however, is how an object becomes perceptible, memorable, intelligible, and thinkable before the subject forms a representation of it. In other words, psychological explanation often begins from the assumption that “the subject is already confronted with certain information,” while paying less attention to how such information has already disclosed itself in some manner before being grasped by the subject.

This question has become particularly important in contemporary psychology and cognitive science. Research on embodied cognition, predictive processing, action-oriented cognition, and active inference has, to varying degrees, moved beyond closed models of internal representation by emphasizing the dynamic coupling between subject and environment, perception–action cycles, and situated cognition (Gibson, 1979; Wilson, 2002; Friston, 2010; Hohwy, 2013; Clark, 2016). The shared contribution of these theories lies in the fact that they no longer understand cognition simply as the passive internal copying of external stimuli. Instead, they emphasize the dynamic structure among organism, body, environment, action, and prediction. Nevertheless, even though these theories have overcome many limitations of traditional representationalism, they still need to further address the following question: how do objects in the environment become open to the subject in a graspable way before subjective perception or representation? If cognition is not an unmediated copy of objects by the subject, what exactly mediates the relation between subject and object?

Recent discussions of the theory crisis in psychology have emphasized that psychological science requires not only methodological refinement, but also stronger conceptual and theoretical foundations. Oberauer and Lewandowsky (2019) argued that psychology faces not only a replication crisis but also a deeper theoretical crisis, namely, that many studies lack theoretical frameworks capable of generating precise, cumulative, and explanatory knowledge. Muthukrishna and Henrich (2019) similarly emphasized that many problems in psychology are not merely methodological but reflect insufficient theoretical development. Van Rooij and Baggio (2021) further argued that psychology needs theories with greater explanatory depth if empirical testing is to become theoretically cumulative. Van Dongen et al. (2025) proposed the notion of productive explanation, which connects phenomena, mechanisms, models, and testable predictions. The present article does not claim to provide a fully operationalized productive explanation, but instead seeks to clarify a prior conceptual condition for future theory-building in psychology.

The need for such conceptual clarification becomes clearer when the problem of object availability is considered across existing approaches. Information-processing, representational, ecological, embodied, and predictive theories have each clarified important aspects of cognition, including input processing, representational content, organism–environment coupling, action, and prediction. Yet these approaches often begin from a relation in which objects are already available to the cognitive system in some form, whether as input, content, affordance, or prediction-relevant structure. What remains less explicitly addressed is the prior question of how an object becomes available to cognition before it is processed, represented, predicted, or acted upon as a determinate psychological object. This residual question motivates the present article’s turn to in-itself information as a conceptual resource for analyzing object-disclosure and informational mediation.

The concept of in-itself information is therefore not introduced here as a ready-made solution, but as a theoretical resource to be reconstructed for psychological purposes. This concept originates from the Chinese philosophy of information. Within this theoretical framework, in-itself information is not meaning already understood by the subject, nor encoded content within a symbolic system. Rather, it is the objective and indirect existence presented by the object before subjective intervention through its structure, state, differential relations, and interactions (Wu and Da, 2021). The question it seeks to answer is not “How does the subject interpret information?” but “How does the object already disclose itself before being interpreted?” In this sense, in-itself information is neither the result of cognitive activity nor the content of subjective representation. It is, instead, the pre-representational condition through which an object becomes open to the subject and thereby becomes a psychological object. The object first discloses itself in the form of in-itself information, after which the subject identifies, selects, processes, and recreates such information through perception, memory, thought, and symbolic activity.

Therefore, this article aims to clarify a fundamental but often implicit premise in psychological science: how a cognitive object is already given in an informational form before entering subjective representation. To address this question, this article first examines the contributions and limitations of information-processing and representational theories in psychology. It then explicates the basic determinations of in-itself information and distinguishes it from semantic information, representational information, and subjective information. Subsequently, the article analyzes the modes through which in-itself information unfolds, including the information field, the processes of informational assimilation and alienation, the three qualities of information, and the logical cycle of informational activity. On this basis, the traditional “subject–object” schema is reconstructed as a “subject–information–object” model. Conceptually, the article argues that the object becomes cognitively accessible through the information it discloses before it is represented or symbolized by the subject. In doing so, it reconfigures the relation between mind and world. The proposed framework offers a foundation for rethinking cognitive generation, psychological objects, representation, and technologically mediated cognition, while addressing the field’s pressing need for stronger theoretical explanation.

## 2 From information processing to informational mediation: an important issue in psychology

The rise of cognitive psychology in the twentieth century was closely associated with the introduction of the concept of information into psychological explanation. Information-processing models understood the cognitive system as a system that receives, encodes, stores, transforms, and outputs information (Neisser, 1967; Newell and Simon, 1972). Perception, attention, memory, and decision-making could all be described as processes of information processing. This information-processing approach enabled psychology to move beyond the limitations of a purely stimulus–response model and to study mental activities that cannot be directly observed in a more operationally tractable manner.

However, in many information-processing models, “information” is often presupposed as an input already available to the subject or system, and the primary focus is placed on the processes of encoding, processing, and output that occur after input has been received. In other words, information-processing psychology explains how the subject processes information, but it does not sufficiently explain how the object discloses itself informationally before being processed by the subject. The issue is not that information-processing theory neglects the external environment, but that it typically treats the availability of input as a starting point rather than as a theoretical problem requiring analysis.

Classical cognitive science generally holds that the cognitive system grasps the external world through internal representations, and that mental processes can be understood as operations performed on these representations (Fodor, 1975; Marr, 1982). This account explains how the subject can remember, reason, plan, and imagine in the absence of external objects. Yet representationalism also faces a fundamental problem: how does a representation acquire its content about an object? If a representation is merely an internal state of the subject, how is its relation to the external object established? Beginning solely from internal representations makes it difficult to adequately explain the generative conditions of psychological objects. Dretske (1983) attempted to explain the foundations of knowledge and representational content through the objective information-carrying relation. His theory already rejects the view that information is merely subjective meaning or an internal mental construction. However, Dretske’s main concern lies in how the information carried by a signal can ground semantic content, belief, and knowledge. The present framework shifts the emphasis from the informational content carried by a signal to the prior disclosure of the object itself. In-itself information is not, in the first instance, concerned with how a signal comes to carry the information that something is the case, but with how an object becomes available as a possible psychological object before such information is stabilized as propositional, semantic, or representational content.

Ecological psychology provides another explanatory path. Gibson (1979) emphasized affordances in the environment and argued that perception is not the result of the subject’s internal processing of stimuli, but a process through which the organism directly grasps possibilities for action in the environment. Embodied cognition and action-oriented cognition further emphasize the constitutive roles of the body, action, and environment in cognition (Thompson, 2007; Chemero, 2009). The distinction between Gibsonian ecological information and in-itself information requires particular clarification. Gibsonian information is not subjective representation and not an internal copy of the world. It is objective, pre-subjective, and available in the ambient array (Gibson, 1979). In-itself information is likewise objective and pre-subjective, but it is proposed as a broader philosophical-psychological account of object disclosure. It refers to source-grounded, receiver-relative differences in an object’s structure, state, relations, and interactions through which the object becomes informationally available across different forms of psychological relevance, including perception, action, memory, symbolic interpretation, and diagnostic judgment. The distinction is therefore primarily one of explanatory scope rather than temporal priority, the explanatory scope of in-itself information is not defined primarily by organism–environment specification for perception and action.

Consider a cup on a table. From a Gibsonian perspective, the cup is specified in relation to possible action: it is graspable, movable, affording drinking, and located within an actionable layout. From the perspective of in-itself information, the same cup discloses itself more broadly through its contour, material, color, spatial position, surface traces, cracks, stains, temperature, and relations to surrounding objects. Some of these features may become affordances, but others may become perceptual objects, memory cues, symbolic meanings, esthetic qualities, or diagnostic signs without being immediately action-relevant. Thus, the concept of in-itself information does not replace Gibsonian ecological information. Rather, it broadens the analysis from information for action to the pre-representational disclosure through which an object becomes available as a possible psychological object.

It can therefore be seen that information-processing theory, representational theory, and ecological psychology have all discussed the relation between information and cognition at different levels. Yet they still need to answer the following question: before being processed, represented, grasped, or predicted by the subject, how is an object already open to the subject in an informational manner? Informational mediation is proposed precisely as a response to this theoretical gap. Informational mediation emphasizes that the subject does not directly encounter a bare object; rather, the object becomes accessible to the subject through the information it discloses. Perception, memory, imagination, and thought do not possess the object itself in an unmediated way. Instead, they involve a series of processes of processing, preservation, transformation, and recreation that unfold between object-information and subjective activity. If future psychology is to further explain the relation between mind and world, it must not only study how the subject internally processes information; it must also examine how the object opens itself to the subject in an informational manner.

## 3 In-itself information as a marker of objective indirect existence

Before introducing the concept in detail, it is necessary to clarify the sense in which the term “in-itself” is used in this article. In Western philosophy, “in-itself” inevitably evokes Kant’s notion of the thing-in-itself, which refers to the object as it is independently of the conditions of possible experience and therefore cannot be directly known as an object of experience (Kant, 1998). The present article does not use “in-itself” in this Kantian sense. In-itself information is not a noumenal object hidden behind appearances, nor does it designate something absolutely inaccessible to cognition. On the contrary, the term refers to the pre-subjective status of information before it has been grasped, interpreted, represented, or symbolically reconstructed by a subject. Thus, “in-itself” here does not mean unknowable in principle; it means not yet subjectively appropriated. It names the object’s informational availability prior to its transformation into semantic content, representation, or psychological meaning.

The theory of in-itself information is grounded in the Chinese philosophy of information, particularly in its account of the essence of information and its classification of informational forms. This theory defines the material world as the world of direct existence and the informational world as the world of indirect existence. In its original philosophical context, this theory proposes a broad account of the domain of existence, often summarized by the formula “existence = matter + information” (Wu, 2005). In the present article, however, this formula is not used as an independently defended metaphysical conclusion. It functions as a background assumption of the Chinese philosophy of information from which the more limited psychological concept of in-itself information is reconstructed. An object can become available to a subject through its structure, state, differential relations, and traces before it becomes semantic content or subjective representation. Within this framework, in-itself information is treated as the marker of objective indirect existence and as the original form of information before it has been recognized and grasped by the subject (Wu et al., 2021).

At the psychological level, in-itself information can be summarized in terms of three determinations. First, in-itself information has object-disclosiveness; that is, it refers to how an object presents itself before subjective interpretation. Second, in-itself information has pre-subjectivity; that is, it does not depend on the subject’s completed acts of meaning-giving or representation. Third, in-itself information has mediacy; that is, it is neither the object itself nor the subject’s representation itself, but the mediating basis through which an object enters into a cognitive relation with the subject.

The importance of in-itself information lies in the fact that it releases information from being confined to subjective representational activity. If information refers only to content that the subject already knows, has already interpreted, or has already symbolized, then information remains subordinate to consciousness, language, or subjective meaning. Such a concept of information cannot explain why an external object can become a cognitive object before the subject has formed a representation of it. The theory of in-itself information proposes that information is, first of all, not “what the subject knows,” but “how the object discloses itself in its existence.” This disclosure does not presuppose the subject’s understanding. Rather, it already occurs within the object’s real existence, differential structure, and interactions.

It is necessary here to distinguish in-itself information from semantic information. Semantic information usually involves meaning, truth value, propositional content, or conditions of interpretation (Floridi, 2011; Lundgren, 2019). It addresses questions such as “What does information mean?” and “How does information acquire meaning within a symbolic system?” In-itself information, by contrast, first addresses the question of how an object discloses itself before being understood. For this reason, in-itself information is closer than semantic information to the pre-subjective condition for the generation of psychological objects.

In-itself information also differs from representational information. Representational information usually means that an internal state of a system represents or refers to an external object. Representational theory focuses on how the subject or cognitive system “represents” the object. In-itself information, however, focuses on how the object “discloses” itself through its own structure and interactions before being represented by the subject. This does not deny the role of representation. Rather, it situates representation within a more fundamental relation of informational mediation. Representation is neither generated out of nothing nor a direct copy of the object itself. It is the result of the subject’s selection, processing, organization, and recreation of the information disclosed by the object. In other words, the occurrence of representation presupposes in-itself information.

Nor is in-itself information purely subjective meaning. Subjective meaning depends on the subject’s needs, language, and interpretive framework. In-itself information, by contrast, emphasizes the object’s disclosive character before subjective meaning-giving. Of course, in-itself information enters psychological reality only after it has been grasped, selected, processed, and symbolized by the subject. Human beings can generate an understanding of objects and situations that they have not directly experienced through mental simulation, which indicates that cognition itself has an evidently constructive and generative character (Gilbert and Wilson, 2007). Yet the pre-subjective character of in-itself information lies in the fact that it is not arbitrarily created by the subject.

The theory of in-itself information resonates with several discussions in contemporary philosophy of information, cognitive science, and information studies. Floridi (2011) developed the philosophy of information as a systematic inquiry into information, knowledge, reality, and meaning. Sprevak (2020) further pointed out that information processing should not be simply equated with representational processing. Jarrahi et al. (2023) also proposed that information has both objective and subjective dimensions. Krzanowski’s (2020) discussion of physical information suggests that the foundation of information need not first appeal to meaning, but can be understood in terms of the organization, structure, and form of physical objects. The theory of in-itself information rejects the complete reduction of information to subjective meaning. By defining information as “objective indirect existence,” it identifies the pre-subjective status of information as a basis for reconstructing the relation between existence and cognition.

In-itself information can therefore enter the core problem domain of theoretical psychology. Psychological science studies mind, behavior, and experience, but the mind does not operate in a vacuum, and behavior is not an internal output detached from the environment. The subject is always related to objects, the body, the environment, and technological systems. An object can enter psychological activity because it first opens itself to the subject in an informational manner. In-itself information is the informational name for this openness.

## 4 The unfolding mechanisms of in-itself information

In-itself information is not a static concept; rather, it has its own mechanisms of unfolding. To explain how an object opens itself to the subject in an informational manner, it is necessary to further analyze the dynamic structure of in-itself information. The information field, together with informational assimilation and alienation, constitutes the two basic forms of in-itself information. The circular logic of informational activity characterizes its mode of operation (Wu and Wang, 2018).

### 4.1 The information field: how an object discloses itself

An information field refers to the mechanism through which an object discloses its mode of existence, state, organization, and relational differences. The article does not assume that an object exists as a bare substrate separable from its organization. In physical cases such as electromagnetic fields or waveguides, the source and its field-structured organization may be inseparable. In the general sense of physics, a field is first of all a material field with a specific matter–energy structure. An object or object-system does not exist in an isolated or static manner, but manifests its mode and state of existence within field relations (Maxwell, 1865). However, this physical premise does not by itself justify the concept of an information field. A material field should not be called an information field merely because it is causally produced by an object. Causation alone is insufficient. The field becomes informational only insofar as its differentiated structure is source-dependent and, in principle, discriminable by a possible receiving system. In this sense, in-itself information is objective in its source-grounding but receiver-relative in its informational availability. This formulation distinguishes three analytically distinct levels: source-dependent physical structure, structure that is in principle discriminable by a possible receiver, and its actual uptake, interpretation, or representation by a particular subject. The first does not require actual perception; the second is receiver-relative but remains pre-subjective insofar as it does not yet involve actual subjective uptake or interpretation; and the third marks the entry of information into psychological activity.

This distinction is crucial. Every information field presupposes some material or energetic carrier, but not every material field is informational in the technical sense relevant here. A causal effect becomes informational when it carries a structured difference through which something about its source can be distinguished, preserved, transmitted, or reconstructed. The term “information field” therefore does not name an entity independent of matter, nor does it redescribe physical causation as information. It refers to the informational function of a material field: the capacity of its differentiated structure to disclose aspects of its source before those aspects are interpreted by a subject. Information should not be regarded merely as transmissible message content; rather, its relation to structure, representational carriers, and the differential character of objects must also be taken into account (Tambassi, 2022).

For example, the light reflected from a cup is a physical process, but it is not psychologically relevant information merely because photons are reflected. It becomes informational when the reflected structure varies with the cup’s contour, color, texture, position, and relation to its surroundings in such a way that these differences can be detected and organized by a visual system. Prior to subjective interpretation, this structured field already makes the cup available as something that can be perceived, recognized, remembered, or symbolically interpreted.

This account of object-disclosing structure also clarifies how the information field differs from, while remaining compatible with, Lewin’s field-theoretical tradition. Lewin showed that psychological events should be understood within a structured person–environment relation, rather than through isolated properties of either the individual or the environment alone (Lewin, 1936, 1943). The information field specified above addresses a narrower condition within this relational view. Before the cup can enter a psychological field as usable, familiar, valuable, or action-relevant, its contour, material, position, traces, and relations must first become available as discriminable object-related information. The information field therefore clarifies how an environmental element becomes available for perception, memory, evaluation, and action before being organized within the subject’s life space.

### 4.2 Informational assimilation and alienation

When an information field acts on another thing—that is, on a receiver—or on a subjective system, the receiver may accept and preserve certain information through changes in its own structure. Correspondingly, when the source derives an information field or interacts with the external world, it also changes its own structure and preserves traces of the information it has previously externalized in the form of object-encoded patterns. This is the process of informational assimilation for the receiver and informational alienation for the source.

In the real world, nothing is absolutely original or completely untouched by interaction; instead, the current structure of any given thing often bears the condensed traces of prior interactions. The current structure of an object is not merely a spatial arrangement; it often preserves informational traces of the process through which it was formed. Geological structures, tree rings, and bodily wrinkles, for example, are all sedimentations of information within structure. There is often a traceable relation between the current structure of a system and its dynamic process of evolution (Murphy et al., 2024; Wang et al., 2024). Research in developmental biology and epigenetics also shows that structure is not merely form, but can preserve the history and regulatory information of a system (Negrete and Oates, 2021; Owen et al., 2023).

At a broad information-philosophical level, assimilation and alienation can describe how interacting systems preserve traces of external influence and externalize aspects of their own states. However, the present article does not treat every causal interaction as psychologically relevant information assimilation or alienation. In the psychological sense intended here, assimilation refers to the process by which a subject, organism, or cognitively organized system incorporates object-related information into its perceptual, mnemonic, bodily, affective, or cognitive organization. Alienation refers to the process by which a subject externalizes its own state, intention, affect, or action into the environment in a form that can be perceived, interpreted, or responded to by others.

Perception can therefore be understood as an initial form of psychological information assimilation. External objects disclose information through light, sound, touch, and contextual structure, and the subject selectively incorporates this information into perceptual and bodily organization. Memory can be understood as the preservation and reorganization of previously assimilated information. It is not a static copy of the past, but a structured reactivation and transformation of traces within the subject. Imagination and prediction further recombine assimilated information in relation to possible futures (Gilbert and Wilson, 2007). Action, expression, and communication, by contrast, are forms of psychological information alienation: through language, gesture, tool use, and social behavior, the subject externalizes its own states, intentions, emotions, and values into forms that can be perceived and responded to by others. Psychological activity is therefore not enclosed within the individual, but unfolds through continuous processes of assimilation, alienation, and reorganization in the subject–object relation.

### 4.3 The circular logic of informational activity

**Figure 1:**
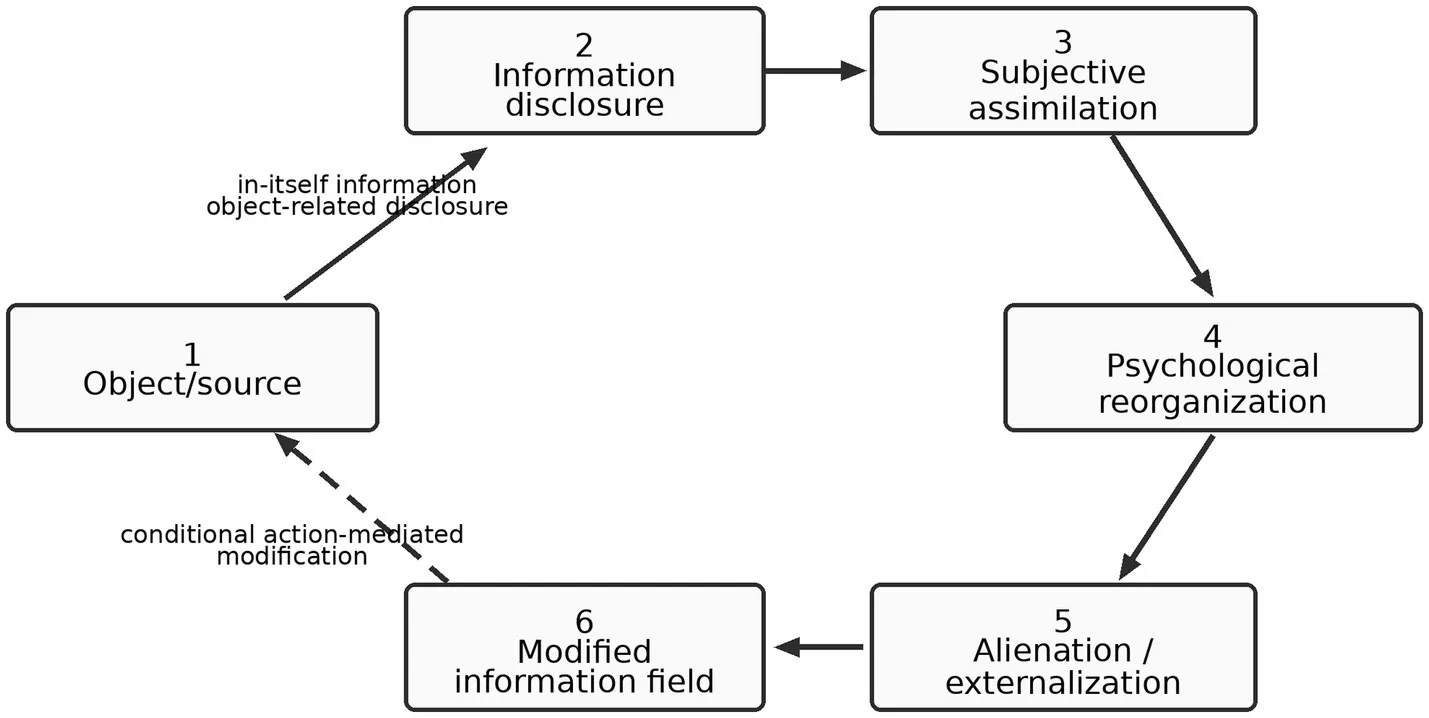
The figure shows the six-stage subject–information–object cycle. In-itself information denotes object-related disclosure before subjective appropriation. The dashed arrow indicates conditional, action-mediated modification, not passive alteration of the material object.

The unfolding of in-itself information is not a one-way transmission from object to subject, but a recursive cycle of informational mediation. As shown in Figure 1, the cycle begins with an object or source that discloses information through its states, structures, relations, and histories. This disclosed information is then selectively assimilated by the subject through perception, memory, affective orientation, and bodily action. Once assimilated, information may be preserved, reorganized, anticipated, or symbolized within psychological activity. Through action, expression, communication, and tool use, the subject externalizes aspects of its own state into the environment, thereby modifying the informational field. This modified field conditions subsequent perception and action, so that the cycle resumes under historically altered conditions rather than simply repeating itself.

Many traditional models of cognition tend to adopt a linear cognitive schema. However, actual cognitive activity is not merely a linear input–output process. The subject’s actions change the environment, and changes in the environment in turn alter the information that the subject can subsequently receive. There is continuous perception–action coupling between subject and environment (Friston, 2010; Stangl et al., 2023). The theory of in-itself information explains that the subject can become circularly coupled with the environment because objects continuously disclose information, the subject continuously assimilates information, and the subject, through action, alienates its own information, thereby changing the new information field. Cognition, memory, action, and social interaction all occur within this circular process.

This circularity should not be understood as an ontological claim that the object is produced by the subject or by the cycle itself. It concerns psychological mediation, namely the way an independently existing source becomes available as a psychological object. The relevant distinction is between the source object, the object-related information disclosed through its structures, states, relations, and traces, and the psychological object formed when such information is taken up within perception, memory, affective orientation, action, and symbolic activity. Subjective activity may reorganize later information fields, but what is transformed is not the object’s existence as such. What changes is the object’s availability, salience, and meaning within an ongoing subject–world relation.

This point also clarifies the sense in which the relation is reciprocal. Passive perception, feeling, or thought does not by itself change the material object. The moon illusion, for example, changes the apparent size of the moon within perceptual experience, but it does not alter the physical moon. The reciprocal relation becomes relevant when cognition is linked to action, tool use, measurement, communication, or technological intervention. Moving a cup, changing illumination, marking a surface, or deploying a sensor can modify the material or informational conditions under which the object is subsequently encountered. Thus, the cycle should be understood as action-mediated and condition-dependent. Thought alone does not transform the material object, but subject-guided action may reorganize the conditions through which the object is disclosed and psychologically taken up.

The subject’s cognition of the world does not begin from a blank state. Rather, it takes place in a world already organized by informational structures, historical traces, relational networks, and technological media. Explanations of reality need to be advanced in a direction that gives greater emphasis to informationality and relationality (Görnitz, 2017). Any current psychological structure should not be understood as an isolated state, but as a structure with historical generativity. Cultural psychology and clinical psychology both show that current psychological objects are often the result of the joint sedimentation of history, body, environment, and symbolic systems (Thompson, 2007; Bailey and Smith, 2024). Thus, a psychological object is not an isolated entity at a single moment, but is generated within the circular process of informational activity. Cognition, likewise, is not a one-time reflection, but a historical, structural, and situated process of generation.

## 5 The three qualitative grades of information and the hierarchical generation of psychological objects

The theory of in-itself information not only emphasizes the objectivity of information, but also further distinguishes among different levels of information. According to the Chinese philosophy of information, information presents three qualitative grades (Wu, 2005).

The first qualitative grade of information is the first-order objective manifestation of direct existence, namely, the direct manifestation of the object’s current structure and state. For example, the shape, color, and temperature of an object, as well as the spatial layout and lighting structure of a scene, are all manifestations of the object’s current state. Perception first encounters this type of information precisely. The subject does not directly enter into the essence of the object, but establishes a primary perceptual relation through the structure and state currently manifested by the object. Li et al. (2023) argue that, compared with the conceptual structure of objects, visual working memory capacity depends more strongly on the perceptual salience of objects. This suggests that the subject’s primary psychological maintenance of objects is first constrained by currently manifested information, such as color and shape.

The second qualitative grade of information is the multi-level objective manifestation of direct existence, namely, the manifestation of historical relations, previous interactions, and indirect traces materially or structurally sedimented in the object or source itself. Its defining feature is that the historical information remains carried by the source, even though its retrieval and interpretation may depend on the capacities and knowledge of a subject. For example, a face or body may preserve observable traces of aging, injury, or previous interaction.

The third qualitative grade of information refers to the creative subjective relations that the subject confers upon information through abstraction, convention, and substitutive representation. These include linguistic and conventional meanings, autobiographical significance, affective valuation, and other relations constituted through abstraction, interpretation, convention, and representation. At this level, information is no longer merely the objective manifestation of an object’s state or historical traces; rather, it is incorporated into symbolic and social relations created by the subject. Concepts and theoretical models in psychological science also belong to the third qualitative grade of information. They are not simple copies of natural objects, but are constructed and stabilized within the symbolic systems, methodological norms, and theoretical commitments of research communities. Recent research has shown that individuals’ memories of familiar people and places can be transformed into subject-specific semantic vector representations and can be tested in the encoding of brain activity. This indicates that after information enters subjective processing, it can indeed acquire new representational forms and functions through language–semantic systems (Bruera and Poesio, 2024).

The theory of the three qualitative grades of information can help us reconceptualize the generation of psychological objects. Representation is not a simple or self-evident concept; rather, its relation to objects, tasks, environments, and interpretive frameworks requires further clarification (Richmond, 2023). Accordingly, the theory of the three qualitative grades of information understands psychological objects as products of a multi-level process of informational generation. Take a “cup” as an example. First-grade information includes the cup’s shape, color, position, and graspable structure. Second-grade information includes wear, scratches, discoloration, repairs, and other historical traces. Third-grade information includes the linguistic concept “cup,” its culturally conventional uses and meanings, as well as autobiographical memories, affective significance, or personal meanings associated with the cup by a particular subject. The “cup” perceived by the subject is not any one of these three levels taken in isolation, but a psychological object formed at their intersection.

The three-grade scheme approaches a different question from familiar level-based or content-based distinctions. Marr’s levels address how a cognitive system may be explained, including the computational, representational-algorithmic, and implementational levels of analysis (Marr, 1982). Levels-of-processing accounts concern the depth of encoding (Craik and Lockhart, 1972), and the perceptual–conceptual distinction separates perceptual features from conceptual structure. By contrast, the three-grade scheme concerns how object-related information becomes layered in the formation of a psychological object. Its added value lies especially in the second grade, understood as historically sedimented and context-bearing traces. A worn cup used for years by a family member, for example, is not psychologically different from a new cup merely because of its current perceptual features or because both fall under the concept “cup.” It also preserves traces of use left by an individual. These traces are neither reducible to immediate perception nor identical with explicit semantic content. Li et al.’s (2023) distinction between perceptual distinctiveness and conceptual structure helps clarify the relative independence of the first and third grades, while Bruera and Poesio’s (2024) work on personally familiar people and places helps illustrate why historically sedimented, autobiographical, and relational information cannot be fully reduced to generic semantic categorization. Thus, the three-grade scheme integrates current perceptual disclosure, historical traces, and symbolic-semantic construction into a single account of psychological object formation.

## 6 From “subject–object” to “subject–information–object”: reconstructing the cognitive schema

The most direct contribution of the theory of in-itself information to psychological science is that it reconstructs the cognitive schema from “subject–object” to “subject–information–object.” Traditional epistemology and many psychological models often understand cognition as the subject’s reflection, image, or representation of the object. Although different theories understand reflection and representation in different ways, they often share an implicit premise: that some form of direct relation can be established between subject and object. The theory of in-itself information, by contrast, argues that the subject does not directly possess the object itself, but enters into relation with the object through the information disclosed by it. Cognition is not an unmediated grasp of the object “itself,” but is always realized through some form of informational connection. In other words, cognition is not a direct contact between subject and object, but a generative process realized through informational mediation (Wu, 2005).

In the “subject–information–object” model, information is neither passively transmitted content nor representational material located within the subject. Rather, it is the mediating dimension through which object and subject enter into relation. Without the informational disclosure of the object, the subject cannot enter the cognitive process. Without the subject’s informational processing, the object’s disclosure cannot become a psychological object. Perception is the subject’s initial recognition of this informational disclosure; memory is the subject’s internal preservation of this informational disclosure; thinking is the subject’s reorganization of this information; and symbolization is the process through which the subject incorporates information into language, concepts, and social relations.

This model contains at least three levels. The first is the level of object disclosure. The object discloses itself through structure, state, differential relations, interactions, and historical traces, thereby forming in-itself information. The second is the level of informational mediation. The information disclosed by the object enters subjective activity through the perceptual system. The third is the level of psychological generation. In the process of processing and symbolizing information, the subject forms psychological objects, representations, and judgments. These three levels are not arranged linearly, but interact within a circular process.

The subject–information–object cognitive schema can help us reconceptualize cognitive outcomes. A cognitive outcome is neither the object itself nor an arbitrary construction by the subject, but the unified result of object-information and subjective processing within a mediating process. This cognitive schema also has methodological significance. If psychology studies only internal variables of the subject, it may overlook the informational structure of the object and environmental mediation. If it studies only external stimuli, it may overlook subjective processing and symbolization. If it studies only behavioral output, it may overlook the circular process of information. Therefore, psychology needs to attend simultaneously to three levels: how the object discloses information, how the subject processes information, and how the two generate psychological objects and action outcomes in concrete situations.

To present this model more clearly, its core propositions can be summarized as follows.

Proposition 1: the object-disclosure proposition. The formation of any psychological object presupposes the informational disclosure of the object. The object does not enter subjective cognition as a bare entity, but opens itself to the subject in the form of in-itself information.

Proposition 2: the informational-mediation proposition. There is no completely unmediated cognitive relation between subject and object. Perception, memory, and thinking all occur within processes of informational mediation.

Proposition 3: the psychological-generation proposition. A psychological object is neither a copy of an external entity nor an arbitrary construction of internal representation, but a relational outcome generated between object-information and subjective processing.

Proposition 4: the hierarchical-generation proposition. A psychological object is usually generated jointly by the current disclosure of the first qualitative grade of information, the historical traces of the second qualitative grade of information, and the symbolized relations of the third qualitative grade of information.

Proposition 5: the circular-regulation proposition. The subject not only assimilates object-information, but also alienates its own information through action, expression, and technological practice, thereby changing subsequent information fields and the generative conditions of psychological objects.

The five propositions do not by themselves constitute fully operationalized predictions, but they specify where the subject–information–object model can be brought into contact with empirical research. Proposition 2 could be examined by varying a specific informational property while holding object identity, overall perceptual quality, and major affordances relatively constant. If wear or repair traces were selectively altered while basic recognition and affordance judgments remained stable, corresponding changes in judgments of prior use, memory, affective evaluation, or diagnostic interpretation would isolate the contribution of object-carried historical information. This selective dissociation would support informational mediation more specifically than the broad observation that changes in presentation affect cognition.

For Proposition 3, the critical comparison is between a generic object–subject interaction model and a model that explicitly incorporates independently operationalized informational variables. The informational-mediation model would have a distinct empirical advantage if those variables explained additional variance or improved cross-validated prediction beyond object features, subject variables, and their interaction. If the generic interaction model performed equally well and the informational variables added no explanatory or predictive value, the distinctive contribution of the subject–information–object framework would be weakened. In this sense, the propositions are not merely terminological reformulations. They are conceptual commitments that can be connected to future empirical designs and model comparisons.

Therefore, the subject–information–object model is proposed as a reconstructive rather than eliminative framework. It does not replace existing theories of perception, cognition, or representation, but clarifies a prior mediating condition that they often presuppose, namely that objects become psychologically available to subjects through informational mediation.

## 7 Discussion: the significance of the theory of in-itself information for future theoretical and philosophical psychology

### 7.1 Significance for psychological explanation

The theory of in-itself information helps reconceptualize the mind–world relation. The mind is not a purely subjective space enclosed within the head, nor is the world a collection of bare entities that directly enter the mind. Rather, informational mediation exists between mind and world. Objects open themselves to the subject through in-itself information, and the subject grasps, processes, and recreates this information through cognitive activity. The mind, therefore, is not an inner theater detached from the world, but a generative system that enters into relation with the world through informational mediation. Likewise, the world is not a passive collection of objects awaiting subjective representation, but an information field that continuously discloses itself through structure, difference, and interaction.

### 7.2 Significance for psychological constructs

The subject–information–object model clarifies why psychological constructs should be treated neither as fixed inner entities nor as merely conventional labels. Psychological constructs are indispensable in psychology, yet they risk reification when constructs such as intelligence, emotion, or personality are treated as self-contained inner substances, and relativism when they are treated as merely linguistic labels or methodological conveniences. De Boeck et al. (2023) rightly emphasize the need to question, define, and measure psychological constructs more precisely. The present framework contributes to this issue by understanding constructs as relationally constituted psychological objects formed through multiple grades of information.

The construct of emotion illustrates this mediating path. At the first grade, emotion involves currently disclosed bodily, expressive, physiological, and situational information, such as facial expression, posture, arousal, tone of voice, and environmental context. At the second grade, it involves historically sedimented information, including prior interactions, relational context, and learned patterns of response. At the third grade, it becomes symbolically and semantically organized through language, cultural categories, self-narratives, measurement categories, clinical classifications, and theoretical models. Thus, emotion is neither a self-contained inner object nor a merely arbitrary label. It is a layered informational organization through which bodily states, histories of interaction, and symbolic interpretation jointly become psychologically meaningful.

### 7.3 Significance for memory, representation, and the generation of psychological objects

The theory of in-itself information also helps reconceptualize memory and representation. Memory is not the simple storage of past objects, but the preservation, activation, and reconstruction of past information within the subject’s structure. Representation is not an arbitrary internal symbol of the subject, but regenerated information produced after the subject has processed the in-itself information of the object. Memory and representation can thus both be situated within the continuous processes of informational assimilation, preservation, reorganization, and recreation. Recent research on real-world event perception and event memory emphasizes that memory is not the storage of isolated stimuli, but the organization and reorganization of structured information within a continuous stream of experience (Bailey and Smith, 2024).

### 7.4 Significance for technologically mediated cognition and psychology in the age of artificial intelligence

The subject–information–object model is especially relevant to technologically mediated cognition and AI. In contemporary psychological life, subjects increasingly encounter objects through technological systems that select, filter, rank, generate, and transform information. Search engines, recommendation systems, wearable devices, learning analytics, and generative AI systems do not merely transmit information from an external world to a passive subject. They participate in the organization of the information field itself by shaping which objects become visible, which features become salient, and which actions become more likely.

This point is particularly important for human–AI interaction. Cukurova (2026) argues that agency in human–AI interaction should be understood as a system-level property rather than as a capacity located only in the individual human or in the AI system. This view fits the present framework. If agency emerges within a human–AI system, then AI is not simply an external tool added after cognition has occurred, but part of the mediating environment through which subjects perceive, evaluate, remember, decide, and act. In this sense, AI systems can function as components of the information field.

AI-mediated systems may participate in several moments of informational mediation. They can shape information disclosure by producing summaries, recommendations, predictions, or generated outputs; guide assimilation by directing attention and modifying salience; influence the reorganization of psychological objects by offering categories, narratives, and interpretations; and support externalization through design, decision-making, communication, and action. These mediating functions are not limited to systems that simply respond as passive tools. Autonomous vehicles and other agentic AI systems show that artificial systems may operate with varying degrees of independence from immediate human direction after an initial prompt, instruction, or design specification. The present framework therefore does not argue that autonomous AI systems are impossible, nor does it attempt to settle debates about full autonomy or existential risk. Its more limited claim is that autonomous and semi-autonomous AI systems can be analyzed as active mediators that sense, select, predict, decide, and intervene in physical or virtual environments. Thus, the central question for future psychology is not only how individuals use technologies, but how technologically organized information fields, including those shaped by autonomous or semi-autonomous AI, transform the conditions under which objects become psychologically available, salient, interpretable, actionable, and meaningful for human agents and human–AI systems.

## 8 Conclusion

The practical consequence of the subject–information–object framework is that psychological research should treat object-availability itself as part of the phenomenon to be explained. For psychological researchers, this means that studies should not examine only the behavior of the subject or the properties of the object. They should also investigate how an object becomes psychologically present through perceptual conditions, contextual traces, symbolic categories, social interaction, or technological mediation. Accordingly, this framework encourages researchers to specify the mediating informational conditions that make perceptual access, memory, judgment, emotion, and representation possible.

A second implication concerns operationalization and modeling. Psychological objects and constructs should be understood as layered informational organizations rather than as isolated inner entities, simple stimulus effects, or merely nominal categories. This encourages researchers to design studies that compare object-centered, subject-centered, and mediation-centered explanations, and to examine how current perceptual information, historical traces, and symbolic-semantic interpretation jointly shape psychological outcomes. In this sense, the framework provides a new perspective for psychological inquiry by requiring researchers to clarify the informational pathways through which subjects encounter, interpret, and transform the world they inhabit.

## References

[ref1] Bailey H. Smith M. E. (2024). Event perception and event memory in real-world experience. Nat. Rev. Psychol. 3, 754–766. doi: 10.1038/s44159-024-00367-0, 40718750 PMC12290826

[ref2] Bruera A. Poesio M. (2024). Family lexicon: using language models to encode memories of personally familiar and famous people and places in the brain. PLoS One 19:e0291099. doi: 10.1371/journal.pone.0291099, 39576771 PMC11584084

[ref3] Chemero A. (2009). Radical Embodied Cognitive Science. Cambridge, MA: MIT Press.

[ref4] Clark A. (2016). Surfing Uncertainty: Prediction, Action, and the Embodied Mind. Oxford: Oxford University Press.

[ref5] Craik F. I. M. Lockhart R. S. (1972). Levels of processing: a framework for memory research. J. Verbal Learn. Verbal Behav. 11, 671–684. doi: 10.1016/S0022-5371(72)80001-X

[ref6] Cukurova M. (2026). Agency as a system property in human-AI interaction in education. Br. J. Educ. Technol. 57, 1065–1070. doi: 10.1111/bjet.70060

[ref7] De Boeck P. Pek J. Walton K. Wegener D. T. Turner B. M. Andersen B. L. . (2023). Questioning psychological constructs: current issues and proposed changes. Psychol. Inq. 34, 239–257. doi: 10.1080/1047840X.2023.2274429

[ref8] Dretske F. I. (1983). Précis of knowledge and the flow of information. Behav. Brain Sci. 6, 55–63. doi: 10.1017/S0140525X00014631

[ref9] Floridi L. (2011). The Philosophy of Information. Oxford: Oxford University Press.

[ref10] Fodor J. A. (1975). The Language of Thought. Cambridge, MA: Harvard University Press.

[ref11] Friston K. (2010). The free-energy principle: a unified brain theory? Nat. Rev. Neurosci. 11, 127–138. doi: 10.1038/nrn2787, 20068583

[ref12] Gibson J. J. (1979). The Ecological Approach to Visual Perception. Boston: Houghton Mifflin.

[ref13] Gilbert D. T. Wilson T. D. (2007). Prospection: experiencing the future. Science 317, 1351–1354. doi: 10.1126/science.1144161, 17823345

[ref14] Görnitz T. (2017). A century of quantum theory: time for a change in thinking: versus the popular belief that material building blocks are the basis of the reality. Found. Sci. 22, 749–762. doi: 10.1007/s10699-016-9497-4

[ref15] Hohwy J. (2013). The Predictive Mind. Oxford: Oxford University Press.

[ref16] Jarrahi M. H. Ma Y. Goray C. (2023). An integrative framework of information as both objective and subjective. J. Inf. Sci. 49, 582–594. doi: 10.1177/01655515211014149

[ref17] Kant I. (1998). Critique of Pure Reason. Translated and edited by Guyer P. Wood A. W. Cambridge: Cambridge University Press.

[ref18] Krzanowski R. (2020). What is physical information? Philosophies 5:10. doi: 10.3390/philosophies5020010

[ref19] Lewin K. (1936). Principles of Topological Psychology. New York, NY: McGraw-Hill.

[ref20] Lewin K. (1943). Defining the “field at a given time.”. Psychol. Rev. 50, 292–310. doi: 10.1037/h0062738

[ref21] Li Q. Chen Z. Sun Q. Li X. (2023). Which factor affects the storage of real-world object information in visual working memory: perceptual or conceptual information? Front. Psychol. 14:1239485. doi: 10.3389/fpsyg.2023.1239485, 37920736 PMC10619856

[ref22] Lundgren B. (2019). Does semantic information need to be truthful? Synthese 196, 2885–2906. doi: 10.1007/s11229-017-1587-5

[ref23] Marr D. (1982). Vision: A Computational Investigation into the Human Representation and Processing of Visual Information. San Francisco: W. H. Freeman.

[ref24] Maxwell J. C. (1865). A dynamical theory of the electromagnetic field. Philos. Trans. R. Soc. Lond. Ser. B Biol. Sci. 155, 459–512. doi: 10.1098/rstl.1865.0008

[ref25] Murphy C. Thibeault V. Allard A. Desrosiers P. (2024). Duality between predictability and reconstructability in complex systems. Nat. Commun. 15:4478. doi: 10.1038/s41467-024-48020-x, 38796449 PMC11127975

[ref26] Muthukrishna M. Henrich J. (2019). A problem in theory. Nat. Hum. Behav. 3, 221–229. doi: 10.1038/s41562-018-0522-1, 30953018

[ref27] Negrete J. Jr. Oates A. C. (2021). Towards a physical understanding of developmental patterning. Nat. Rev. Genet. 22, 518–531. doi: 10.1038/s41576-021-00355-7, 33972772

[ref28] Neisser U. (1967). Cognitive Psychology. New York: Appleton-Century-Crofts.

[ref29] Newell A. Simon H. A. (1972). Human Problem Solving. Englewood Cliffs, NJ: Prentice-Hall.

[ref30] Oberauer K. Lewandowsky S. (2019). Addressing the theory crisis in psychology. Psychon. Bull. Rev. 26, 1596–1618. doi: 10.3758/s13423-019-01645-2, 31515732

[ref31] Owen J. A. Osmanović D. Mirny L. A. (2023). Design principles of 3D epigenetic memory systems. Science 382:eadg3053. doi: 10.1126/science.adg3053, 37972190 PMC11075759

[ref32] Richmond A. (2023). Commentary: investigating the concept of representation in the neural and psychological sciences. Front. Psychol. 14:1259808. doi: 10.3389/fpsyg.2023.1259808, 37965667 PMC10641478

[ref33] Sprevak M. (2020). Two kinds of information processing in cognition. Rev. Philos. Psychol. 11, 591–611. doi: 10.1007/s13164-019-00438-9

[ref34] Stangl M. Maoz S. L. Suthana N. (2023). Mobile cognition: imaging the human brain in the ‘real world’. Nat. Rev. Neurosci. 24, 347–362. doi: 10.1038/s41583-023-00692-y, 37046077 PMC10642288

[ref35] Tambassi T. (2022). On the informativeness of information system ontologies. Philosophia 50, 2675–2684. doi: 10.1007/s11406-022-00558-0

[ref36] Thompson E. (2007). Mind in Life: Biology, Phenomenology, and the Sciences of Mind. Cambridge, MA: Harvard University Press.

[ref37] van Dongen N. van Bork R. Finnemann A. Haslbeck J. M. B. van der Maas H. L. J. Robinaugh D. J. . (2025). Productive explanation: a framework for evaluating explanations in psychological science. Psychol. Rev. 132, 311–329. doi: 10.1037/rev0000479, 39023936

[ref38] van Rooij I. Baggio G. (2021). Theory before the test: how to build high-verisimilitude explanatory theories in psychological science. Perspect. Psychol. Sci. 16, 682–697. doi: 10.1177/1745691620970604, 33404356 PMC8273840

[ref39] Wang J. Zhang Y.-J. Xu C. Li J. Sun J. Xie J. . (2024). Reconstructing the evolution history of networked complex systems. Nat. Commun. 15:2849. doi: 10.1038/s41467-024-47248-x, 38565853 PMC10987487

[ref40] Wilson M. (2002). Six views of embodied cognition. Psychon. Bull. Rev. 9, 625–636. doi: 10.3758/BF03196322, 12613670

[ref41] Wu K. (2005). Philosophy of Information: Theory, System and Method. Beijing: The Commercial Press.

[ref42] Wu K. Brenner J. Wu T. Wang J. (2021). Information Philosophy: Basic Theory and Interpretation of its Significance. Beijing: Central Compilation and Translation Press.

[ref43] Wu T. Da K. (2021). The Chinese philosophy of information by kun Wu. J. Doc. 77, 871–886. doi: 10.1108/JD-06-2020-0110

[ref44] Wu K. Wang Z. (2018). Natural philosophy and natural logic. Philosophies 3:27. doi: 10.3390/philosophies3040027

